# Surgical Outcome of Decompression and Fixation of Degenerative Lumbosacral Spondylolisthesis Surgery in Pakistani Population

**DOI:** 10.7759/cureus.5493

**Published:** 2019-08-26

**Authors:** Muhammad Tahir, Lal Rehman, Iram Bokhari, Syed Ijlal Ahmed, Ali Afzal

**Affiliations:** 1 Orthopaedics, Jinnah Postgraduate Medical Center, Karachi, PAK; 2 Neurosurgery, Jinnah Postgraduate Medical Centre, Karachi, PAK; 3 Neurology, Liaquat National Hospital and Medical College, Karachi, PAK

**Keywords:** spondylolisthesis, myerding classification, oswestry disability index

## Abstract

Background

Spondylolisthesis is characterized by the slipping of one vertebra, compared with the underlying one, due to structural and degenerative changes. Its origin is multifactorial which includes disc degeneration, facet joint anatomic orientation, iliolumbar configuration, and ligament hyperlaxity. The most common operative treatment is decompression and may require an individualized surgical plan. However, only decompression may progress the slippage which can result in pain or recurrence of neurological complaints. Therefore, lumbar fusion and fixation are considered appropriate to stabilise the spine and prevent delayed deterioration. The aim of our study was to find out the outcome of posterior decompression, with reduction and fixation of lumbosacral spondylolisthesis by Oswestry Disability Index (ODI) to improve further our results.

Methods

This study was conducted from July 2013 to February 2017 including 94 patients with lumbosacral spondylolisthesis. The Meyerding classification was used to grade the extent of vertebral slippage. The assessment was done using the ODI.

Results

There were 50 (53.19%) males and 54 (46.80%) females with a mean age of 44 years ± 10.49 SD. Backache was present in all patients and claudication in 85 (90.42%) patients. There were 10 (10.63%) patients with spondylolisthesis at L3-L4, 36 (38.29%) at L5-S1 and 48 patients (51.06%) at L4-L5 level. In 48 patients with L4-L5 level, 38 (79.16%) were in grade II while six (12.5%) were in grade III. According to the preoperative ODI score, 38 patients were placed in moderate disability, 42 patients were severely disabled while four patients were disabled.

Good outcome was achieved in a total of 79 (84.04%) patients. In 40 (42.55%) patients, with complete reduction, the good outcome achieved in 35 (83.33%) while in 22 (23.40%) patients there was no reduction and a good outcome was achieved in 17 (77.27%) patients. In 38 (40.42%) patients with moderate disability, 32 (84.04%) patients had a good outcome. Post-operative cerebrospinal fluid (CSF) leak occurred in five (5.31%) and wound infection in seven (7.44%) patients while there was no mortality.

Conclusion

Reduction with decompression can have a good outcome in spondylolisthesis, and ODI should be used as a predictor of outcome. It also shows that proper decompression is required and not a complete reduction.

## Introduction

Spondylolisthesis is characterized by the slipping of one vertebra, compared with the underlying one, due to structural and degenerative changes [1]. Its diagnosis is confirmed by a combination of clinical examination and radiological assessment including X-rays, MRI and CT scan. Its origin is multifactorial which includes disc degeneration, facet joint anatomic orientation, iliolumbar configuration, and ligament hyperlaxity [2]. Multiple classification systems have been proposed for this pathology but commonly used classifications are dysplastic, isthmic, degenerative, traumatic, and pathological [3]. Patients complain of pain which is exacerbated by repetitive extension, rotation, return from a flexed position, trivial activities; and relieved by rest [4].

The most common operative treatment is decompression and may require an individualized surgical plan [5]. However, only decompression may progress the slippage which can result in pain or recurrence of neurological complaints. Therefore, lumbar fusion and fixation are considered appropriate to stabilize the spine and prevent delayed deterioration. Outcome after surgery is assessed by a large variety of methods that include Oswestry Disability Index (ODI), Visual Analogue Scale (VAS), radiographs and Beaujon functional score. This study was conducted to find out the outcome of posterior decompression, with reduction and fixation of lumbosacral spondylolisthesis by using ODI index to improve further our results.

## Materials and methods

This descriptive study was conducted between July 2013 and February 2017 with the consent of the patients and approval of the institutional review board with a follow-up of six months to three years. There was a total of 94 patients with lumbosacral spondylolisthesis. This included patients of either sex, aged between 15 and 70 years, grade I spondylolisthesis with disc herniation and grade II and above. Those with traumatic or pathological aetiology, below the age of 15 years, above the age of 70 years, grade I without disc herniation, previously operated, and those with associated cervical or knee problems were excluded. After taking history and doing a clinical examination, a preoperative radiological assessment was done by using X-rays anterioposterior and lateral views, MRI and CT scan with the 3-D reconstruction of the lumbosacral spine. The Meyerding classification was used to grade the extent of vertebral slippage, showing grade I with 0-25% slippage; grade II with 25-50% slippage; grade III with 50-75% slippage and grade IV with 75-100% slippage. The assessment was done by using ODI score which has a questionnaire including 10 components. The score obtained by patients was recorded, and ODI score was calculated as, score achieved by the patient divided by the total possible score, multiplied by 100. According to ODI score, 0% to 20% is a minimal disability, and patients can cope with living activity, 21%-40% is moderate disability and patients are with pain in daily activity and are disabled from work. In severe impairment with score 41%-60%, everyday activity of patients is affected, and they need detailed investigations, while 61%-80% is crippling back pain in which all aspects of life are pinged and with a score of 81%-100%, patients are bed-bound. All patients underwent a surgical procedure in a prone position. We did a decompressive laminectomy, transpedicular screws insertion with rods, reduction and posterolateral grafting from the iliac bone. Discectomy was done in all cases of grade I with disc herniation and drain was inserted in all patient for 24 hours. Postoperatively, X-rays were taken, and ODI score was calculated in follow-up, and it was labelled as good when there was an improvement of more than 20 score and fair when improvement was between 10 and 20 score, and it was poor when improvement was less than 10 score.

Data were analysed by using SPSS, Version 22 (IBM Corp., Armonk, NY). Percentage and frequency were detected, chi-square test and paired t-test were applied, and p-value of less than 0.05 was considered significant.

## Results

Majority of patients were male, 50 (53.19%), as compared to females, 44 (46.80%). Age ranged from 15 to 70 years with a mean 44 years ± 10.49 SD. Backache was present in all patients, claudication in 85 patients (90.42%), straight leg raise (SLR) restricted in 40 patients (42.55%), lumbar extension painful in 87 patients (92.55%). According to the preoperative ODI score, 38 (40.42%) patients (average ODI score 32.63) were placed in moderate disability, 52 (55.31%) patients (ODI score 52.58%) were in severe disability while four (4.25%) patients (ODI score 6.7%) were crippled, as shown in Table 1.

**Table 1:** Pre-operative Oswestry Disability Index (ODI) along with the level of spondylolisthesis

		I	II	III
Moderate (n = 38) (40.42%)	Good (n = 32) (84.21%)	06	26	00
	Fair (n = 4) (10.52%)	02	02	00
	poor (n = 2) (2.63%)	0	02	00
Severe (n = 52) (55.31%)	good (n = 45) (86.53%)	01	36	08
	Fair (n = 3) (5.76%)	01	01	01
	Poor (n = 4) (7.69%)	01	01	02
Severe crippling pain (n = 4) (4.25%)	Good (n = 2) (50%)	01	01	00
	Fair (n = 1) (25%)	00	00	01
	Poor (n = 1) (25%)	00	00	01
Total	94	12 (12.76%)	49 (52.12%)	13 (13.82)

There were 10 (10.63%) patients with spondylolisthesis at level L3-L4, 36 (38.29%) patients at L5-S1 and 48 (51.06%) patients at L4-L5 level. In 48 patients with L4-L5 level, 38 (79.16%) were in grade II while six (12.5%) were in grade III as shown in Table 2.

**Table 2:** Pre-operative grades according to the level of spondylolisthesis

Level	Grades of spondylolisthesis				
	Grade I	Grade II	Grade III	Grade IV	Total patients
L3-L4, n = 10 (10.6%)	03 (30%)	07 (70%)	00	00	10
L4-L5, n = 48 (51.06%)	04 (8.33%)	38 (79.16%)	06 (12.5%)	00	48
L5-S1, n = 36 (38.29%)	03 (8.33%)	25 (69.44%)	08 (25%)	00	36
TOTAL	10 (10.6%)	70 (74.46%)	14 (14.89%)	00	94

There were 10 patients (10.63%) in grade I and 70 patients (74.46%) in grade II. In patients with grade I spondylolisthesis, the complete reduction was achieved in eight patients (80%). However, in 70 patients (74.46%) with grade II, the complete reduction was achieved in 30 (42.85%) patients while there was no reduction in 20 patients (28.57%), as shown in Table 3. Good outcome was achieved in 79 (84.04%) patients. In 40 (42.55%) patients with fair and poor ODI grades, there was complete reduction and the good outcome achieved in 35 (87.5%) while in 22 (23.4%) patients there was no reduction and a good outcome was achieved in 17 (77.27%) patients (Table 4). In 38 (40.42%) patients with moderate disability, 32 (84.21%) patients had a good outcome with six patients (6.38%) were in grade I, and 26 patients (27.65%) were in grade II as shown in Table 1.

**Table 3:** Post-operative reduction of slippage

Pre-operative	Post-operative					
	Complete reduction	I < 25% slippage	II (25-50%)	III (50-75%)	Total	P-value
Grade I <25%, n = 10 (10.6%)	8 (80%)	2 (20%)	0	0	10	0.005
Grade II (25-50%), n = 70 (74.46%)	30 (42.85%)	20 (28.57%)	20 (28.57%)	0	70	0.009
Grade III (50-75%), n = 14 (14.89%)	02 (14.28%)	08 (57.14%)	04 (28.57%)	0	14	0.007
Total = 94	40 (42.55%)	30 (31.91%)	24 (25.53%)	0	94	

**Table 4:** Post-operative scoring according to the level of reduction

Level of reduction	Post-operative ODI score			P-value
	Good	Fair	Poor	
Complete reduction (n = 40)	35 (87.5%)	03 (7.5%)	02 (5%)	0.002
Incomplete reduction (n = 32)	27 (84.3%)	03 (9.37%)	02 (6.25%)	0.006
No reduction (n = 22)	17 (77.3%)	02 (9.09%)	03 (13.63%)	0.004

Post-operative cerebrospinal fluid (CSF) leak occurred in five (5.31%) patients, wound infection in seven (7.44%) patients, urinary incontinence in one patient (1.06%), partial foot drop in one (1.06%) patient, deep venous thrombosis in four (4.25%) patients and paralytic ileus in three (3.19%) patients. There was no mortality noted in our series of patients. All complications improved with conservative treatment.

## Discussion

Spondylolisthesis, a complex and challenging multifactorial condition, shows forwards slippage of one vertebra over another. It can occur at any age and one study shows the average age of 42 years [6]. It may occur both in male and female and one study shows a male to female ratio of 1.1:1 [7]. Degenerative spondylolisthesis occurs mostly at the L4-5 level as opposed to isthmic spondylolisthesis, which occurs most often at the lumbosacral level (L5-S1) [8]. Women demonstrated a significantly higher prevalence of degenerative spondylolisthesis compared to men, with a male-to-female ratio of 1:3, which was also found in our study. Because lower back pain and impaired abdominal muscle function are common during pregnancy and post-partum, resulting in poor spinal mechanics, could be a factor in the development of degenerative aetiology in women [9]. By comparison, in our study, there were more males (54) than females (40) while the average age was 44 ± 10.49.

Patients complain of pain that worsens with activity, usually exacerbated by repetitive extension, rotation, and return from a flexed position, while relieved by rest. In some cases, patients may report radicular symptoms in one or both legs [1]. Thus pain is the predominant feature which was also confirmed in all of our patients. Classification of patients with low back pain into clinical subgroups is considered as being important. Instability is commonly considered a subgroup of chronic lower backache, and the recurrent pain in such patients of spondylolisthesis is thought to be due to abnormal segmental movement.

Radiologically, spondylolisthesis can be described according to its degree of severity, with one commonly used description being grade-I least advanced, and grade-V being most advanced. Surgical indications include progressive slip, significant lumbosacral kyphotic deformity, neurologic deficit, intractable back pain, and refractory radicular pain [10,11].

In one study, there were 29 patients with grade II out of 36 comprising 80.55% and in our study grade II was in 70 patients comprising 74.55% [12]. The slip grade as per Myerding grades was I in 31 (32.29%), II in 39 (40.62%), III in 19 (19.79%), IV in five (5.2%) and two (2.08%) had spondyloptosis [12]. Slippage of vertebrae can occur at any level, but it is more common in the lower lumbar region. In one study, there were 28 patients out of 40 at the L4/L5 level, eight patients at the L5-S1 level and two patients at L3-L4 level while in our study L4/L5 was also the prominent level [13].

Treatment of spondylolisthesis involves both surgical and non-surgical options. Non-surgical treatment is focused on reducing pain, facilitating fracture healing, and preventing any additional vertebral mal-alignment. These non-surgical options can be used alone or in various combinations [14]. Surgical treatment may be necessary if pain persists after extensive conservative treatment and disease progression. Surgery aims at reducing pain and bring the vertebrae back into proper alignment and stabilize the spine to prevent further disability. Treatment options for symptomatic spondylolisthesis continue to be discussed among spine professionals, but recent studies have shown that surgical procedures provided a better improvement in pain and function compared to usual non-operative care [15].

An assessment of the history of surgeries for spondylolisthesis indicates that most surgeons tend to perform spinal procedures through a posterior approach due to more familiarity with this approach, decreased risk of injury to great vessels or vital organs, greater ease of revision operations, ability to operate on multi-levels, and no need for assistance from a general or vascular surgeon [16]. Thus the standard surgical treatment for this disorder with lumbar stenosis is lumbar fusion after standard laminectomy and this strategy is widely adopted, especially in patients with advanced-stage [17]. We have operated our patients through a posterior approach. We have done posterior decompression with reduction and fixation, followed by post-operative radiological and clinical assessment, as seen in studies conducted by other researchers [18]. Another study indicated that circumferential fusion (360°) was associated with greater relief of nerve root pain and better lordosis recovery after one year compared to postero-lateral fusion [19].

There are different scoring systems like ODI, VAS, and Beaujon functional score, etc. but we have used ODI. The ODI is used by clinicians and researchers to quantify disability for low back pain. It is thus currently considered as the gold standard for measuring the degree of disability and estimating the quality of life in a person with low back pain [20]. However, it can be used to assess surgical outcome in patients with spinal surgeries.

Regarding this particular study, while keeping all these parameters in mind, we used this index to measure the symptomatic outcome in our series of patients after surgery. The results indicated good results when assessed by this score after surgery similar to other studies. According to the pre-operative ODI score was 53.7 (±13.1) which improved to 22.5 SD 15.5 at two years follow-up [12]. The average pre-operative ODI score was 51.4, which improved to 18.6 postoperatively [6], but we have divided our patients into three groups with ODI score of 32.63 in moderate disability group while 67 in the group with crippling pain.

In spondylolisthesis surgery, decompression and reduction both are important, but reduction shows no correlation to the clinical outcome [21]. In our study, there is no significant difference between the groups with full reduction and partial reduction as 35 (87.5%) showed the good result with complete reduction out of 40, p-value 0.002, and 27 (84.3%) out of 32, p-value 0.006, showed good results with incomplete reduction.

Like other surgeries, complication can be expected, and there are chances of CSF leak, infection implant failure and neurological deterioration. The long duration of surgery can be a risk factor for superficial or deep wound infection [22]. This could also be an explanation for the development of infection in our group of patients. It is recommended that a reduction in neurological complications may be obtained with constant use of intraoperative neuromonitoring especially in surgical procedures at high neurological risks like spondylolisthesis reduction surgery [23]. In one study, there were a total of 45 patients operated, but there were two cases with implant failure and one case with wound infection but no neurological deterioration [24]. In another study, CSF leak occurred in two patients [25], while in our study CSF leak occurred in five patients. There was no implant failure, and no mortality noted in our series. All our patients with post-operative complications improved with conservative treatment.

In a meta-analysis, authors concluded that fusion with decompression surgery is a better technique when compared to decompression alone for spinal stenosis in terms of the ODI and the VAS for pain. Decompression with fusion is a 3.5-time better surgical technique than decompression alone for spinal stenosis [26].

## Conclusions

Spondylolisthesis is a significant spinal problem, and reduction with decompression can have a good outcome, and ODI should be used as a predictor of outcome. It also shows that proper decompression is required and not a complete reduction.
